# Iatrogenic Botulism Following Cosmetic Botulinum Toxin Injection During a 2025 United Kingdom Outbreak: A Case Report

**DOI:** 10.7759/cureus.117716

**Published:** 2026-10-10

**Authors:** Ellie Keeling

**Affiliations:** 1 Renal Transplant, University Hospitals of Leicester NHS Trust, Leicester, GBR

**Keywords:** adverse drug reaction, botulinum neurotoxin, botulism antitoxin, bulbar weakness, cosmetic injections, cosmetic procedures, iatrogenic botulism, ptosis

## Abstract

Iatrogenic botulism is a rare but potentially life-threatening complication of cosmetic botulinum neurotoxin (BoNT) administration. This report describes a 30-year-old woman who developed progressive neurological symptoms following cosmetic BoNT injections during the 2025 outbreak of iatrogenic botulism in England. She initially presented with headache, facial numbness, throat discomfort, and paraesthesia, with reassuring clinical observations and neurological examination. Over the following days, she developed bilateral ptosis, diplopia, dysarthria, dysphagia, dry mouth, and generalized weakness, prompting re-presentation approximately nine days after injection. The evolving ocular and bulbar syndrome following recent BoNT exposure raised clinical suspicion of iatrogenic botulism. Critical care and difficult airway teams were involved, and serial respiratory monitoring, including forced vital capacity assessment, was undertaken. Botulism antitoxin was administered following specialist advice, with subsequent neurological improvement and no progression to respiratory failure. Serum for botulinum toxin testing was obtained only after antitoxin administration, limiting optimal laboratory confirmation. Neurological examination subsequently returned to normal, although residual nausea and reduced oral intake persisted. This case highlights the potential for iatrogenic botulism to evolve after an initially reassuring presentation, the importance of respiratory surveillance in patients with bulbar symptoms, and the need to obtain diagnostic serum before antitoxin administration without delaying clinically indicated treatment.

## Introduction

Botulism is a rare neuroparalytic syndrome caused by botulinum neurotoxins, which inhibit presynaptic acetylcholine release at peripheral cholinergic nerve terminals. Although classically associated with foodborne, wound, and intestinal forms of disease, botulism may also occur following therapeutic or cosmetic administration of botulinum neurotoxin (BoNT). In iatrogenic botulism, systemic effects extend beyond the intended injection site and can produce cranial neuropathies, bulbar dysfunction, symmetrical descending weakness, and, in severe cases, respiratory failure [1,2]. In contrast, common adverse effects from BoNT procedures are local and transient, including injection-site pain, swelling and erythema [1]. 

Cosmetic BoNT procedures are widely performed and systemic botulism is uncommon, but recognition is important because early manifestations may be non-specific and the neurological syndrome can evolve over several days. Ptosis, diplopia, dysarthria, dysphagia, dry mouth, and generalized weakness are characteristic features, while respiratory muscle involvement represents the major life-threatening complication [1,2]. Diagnosis is primarily clinical and is supported by a compatible exposure history; laboratory confirmation can be difficult in iatrogenic disease and should not delay treatment when suspicion is high [1,2].

In 2025, England experienced an outbreak of iatrogenic botulism associated with cosmetic BoNT procedures. Forty-one clinically confirmed cases were reported across five English regions between June 4 and August 6, 2025; 32 patients required hospital admission and eight required intensive care [3]. A subsequent investigation of a North East England cluster identified an association with an unlicensed BoNT-containing product, highlighting the potential public health consequences of unregulated cosmetic toxin use [4].

This report describes a case occurring during this outbreak in a 30-year-old woman who developed progressive ocular and bulbar neurological manifestations following cosmetic BoNT injections. The case illustrates the diagnostic challenge of an initially non-specific presentation, the importance of respiratory surveillance, and the practical implications of obtaining diagnostic serum before administration of botulism antitoxin.

## Case presentation

A 30-year-old woman presented to the emergency department (ED) with progressive neurological symptoms following cosmetic BoNT injections. She had undergone injections to the forehead, periocular, and perioral regions two days earlier (day -2). The brand, preparation, and total dose administered were unknown. She had previously received cosmetic BoNT injections on five to six occasions without complication but had recently changed her treatment provider.

On day zero, she attended the ED reporting headache, facial numbness, throat discomfort, lower-limb paraesthesia, and a sensation of feeling “drunk.” She was clinically well and haemodynamically stable, with an early warning score (EWS) of zero. Her airway was patent, gait was normal, and neurological examination demonstrated no focal deficit or evidence of respiratory compromise. She was discharged with safety-netting advice.

Over days one to six, her symptoms progressed, with the development of blurred vision and diplopia, bilateral ptosis, altered and slurred speech, dry mouth, dysphagia, and generalized weakness. She also experienced persistent headache, nausea and vomiting with reduced oral intake, and intermittent extremity paraesthesia. On day seven, approximately nine days after the injections, she re-presented to the ED because of worsening symptoms. The temporal evolution of her presentation is summarised in Figure 1.

**Figure 1:**
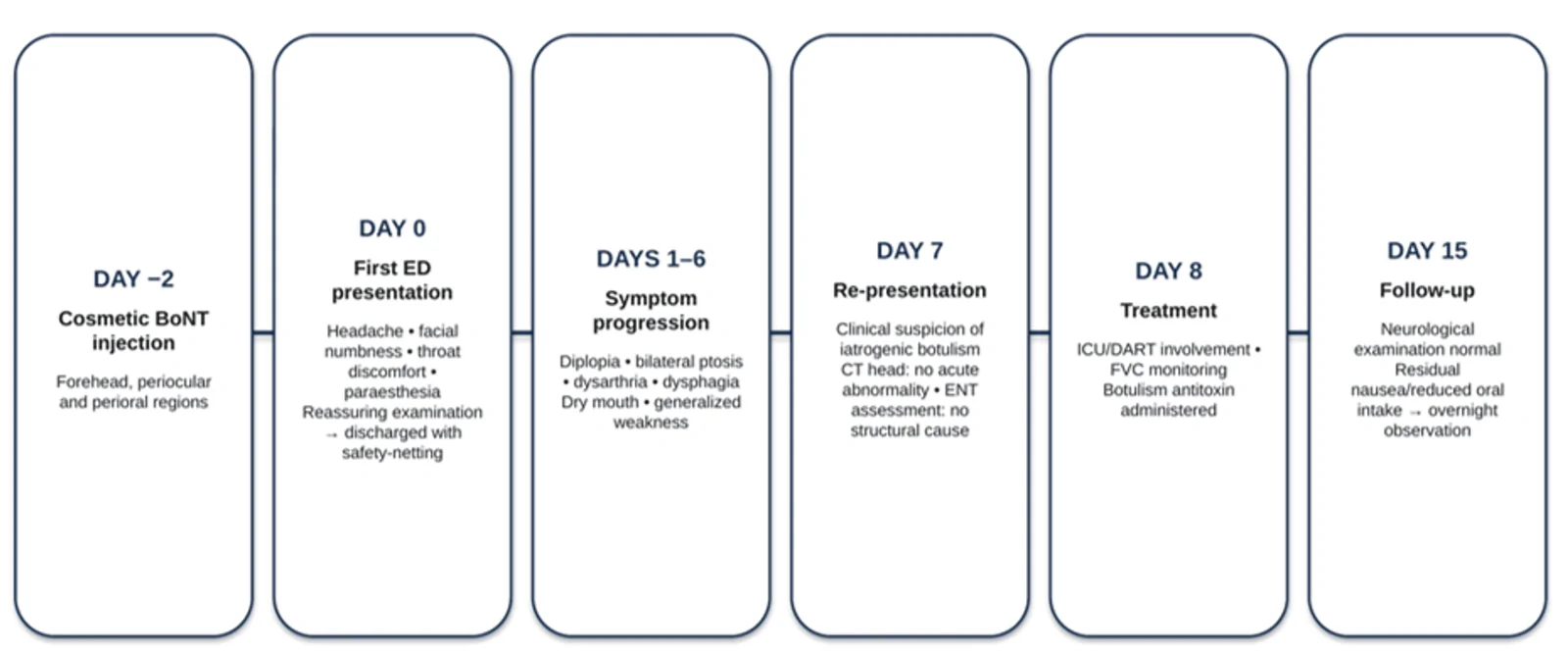
Timeline of clinical presentation, investigation, treatment, and recovery BoNT: botulinum neurotoxin; ED: emergency department; CT: computed tomography; ENT: ear, nose, and throat; ICU: intensive care unit; DART: difficult airway response team; FVC: forced vital capacity. Image credit: Created by the author using Microsoft PowerPoint (Microsoft Corp., Redmond, WA, USA).

On re-assessment on day seven, she remained alert and haemodynamically stable. Neurological examination demonstrated bilateral ptosis, diplopia, reduced forehead movement, altered speech and voice, and dysphagia. Pupils were 3 mm, equal and reactive to light. The remainder of the cranial nerve examination was unremarkable. Upper- and lower-limb reflexes were normal, sensation in tact and formal limb power was 5/5 throughout without a focal motor deficit. She reported increased sensitivity to touch bilaterally over the head. There was no glossal oedema and the uvula appeared erythematous and swollen but was not deviated. 

A non-contrast CT scan of the head demonstrated no acute intracranial abnormality. Routine laboratory investigations were largely unremarkable, as summarised in Table 1.

**Table 1:** Routine laboratory investigations eGFR: estimated Glomerular Filtration Rate.

Parameter	Patient result	Reference range	Units
Haemoglobin	125	120–150	g/L
White cell count	6	4.0–10.0	×10⁹/L
Platelet count	213	150–410	×10⁹/L
C-reactive protein	<5	<5	mg/L
Sodium	141	133–146	mmol/L
Potassium	4.4	3.5–5.3	mmol/L
Creatinine	59	49–90	μmol/L
eGFR	>90	≥90	mL/min/1.73 m²

These findings were reassuring with respect to major metabolic, electrolyte and systemic inflammatory abnormalities but were not considered sufficient to exclude alternative neurological diagnoses.

Given her dysphagia and altered voice, the ear, nose and throat (ENT) team performed flexible nasendoscopy. This demonstrated a patent upper airway, freely mobile vocal cords, and no obstructing lesion or other structural abnormality to account for her symptoms, however, no imaging was saved. 

The combination of recent BoNT exposure and progressive ocular and bulbar neurological features raised clinical suspicion of iatrogenic botulism. The intensive care and difficult airway response teams were involved because of the risk of progression to respiratory muscle weakness. Serial respiratory assessment, including forced vital capacity monitoring, was undertaken, with plans for arterial blood gas analysis and escalation to critical care should respiratory deterioration occur.

Following discussion with the National Poisons Information Service (NPIS) and specialist teams, botulism antitoxin was urgently obtained and administered on day eight. Pyridostigmine 30 mg four times daily was also initially commenced for symptomatic management. A serum specimen for botulinum toxin testing was obtained only after antitoxin had been administered. The eventual assay result was not available in the hospital electronic record and could not be obtained retrospectively; therefore, laboratory confirmation of botulinum toxin cannot be reported in this case.

Her neurological symptoms subsequently improved without progression to respiratory failure or requirement for invasive ventilatory support. Following further specialist advice from the UK Health Security Agency (UKHSA), pyridostigmine was discontinued as it was not recommended. Her respiratory status was monitored following withdrawal without evidence of deterioration.

Nerve conduction studies, repetitive nerve stimulation and single-fibre electromyography were not performed. The diagnosis remained clinical following specialist review, and neurophysiological investigation was not subsequently pursued because of the compatible exposure history and clinical phenotype together with subsequent improvement.

At clinical review on day 15, her neurological assessment had returned to normal, with no respiratory compromise. However, residual nausea and reduced oral intake persisted, necessitating overnight observation and supportive management. There was no further vomiting or neurological or respiratory deterioration. She was subsequently discharged with antiemetic therapy and safety-netting advice. Specialist advice was that residual symptoms could take approximately four weeks from the initial injection to resolve fully.

## Discussion

Iatrogenic botulism results from systemic effects of BoNT administered for therapeutic or cosmetic purposes. BoNT inhibits presynaptic acetylcholine release, producing a characteristic neurological syndrome involving cranial nerve dysfunction followed by symmetrical descending weakness [1,2]. UKHSA guidance describes dysphagia, dysarthria or altered voice, muscle weakness, ptosis, blurred vision, and diplopia among the manifestations of iatrogenic botulism [1]. Symptoms commonly develop several days after injection, although the interval between exposure and clinical manifestations can vary. The temporal relationship between BoNT exposure and the evolution of bilateral ocular and bulbar dysfunction in this patient was therefore strongly supportive of the clinical diagnosis.

An important feature of this case was the relatively non-specific initial presentation. Headache, facial numbness, throat discomfort, and paraesthesia in an otherwise clinically stable patient did not initially produce the characteristic picture of botulism. However, similar symptoms were observed during the 2025 North East England outbreak: headache was reported in 48% of cases, while numbness, tingling, chilling, or burning sensations were reported in 44% [4]. The subsequent development of bilateral ptosis, diplopia, dysarthria, and dysphagia produced a considerably more characteristic neurological syndrome. This demonstrates the importance of appropriate safety-netting and re-assessment when symptoms following cosmetic BoNT exposure evolve beyond expected local adverse effects.

The differential diagnosis of ocular and bulbar weakness includes myasthenia gravis, Guillain-Barré syndrome (GBS) and its variants, brainstem pathology, other neuromuscular junction disorders, and toxic or metabolic causes [2,5]. In this case, myasthenia gravis was considered less likely because of the close temporal relationship with recent BoNT exposure and accompanying dry mouth and sensory symptoms. However, it was not formally excluded by antibody testing or neurophysiology. GBS and its variants were less likely given preserved limb power and normal reflexes, without progression to ascending or generalised flaccid paralysis; however, cerebrospinal fluid analysis and neurophysiological studies were not performed. The non-contrast CT imaging of the head demonstrated no acute intracranial abnormality but, importantly, could not exclude brainstem, demyelinating or neuromuscular pathology and was not considered diagnostic for botulism. The evolving bilateral ocular and bulbar syndrome, characteristic autonomic symptom of dry mouth, recent BoNT exposure and contemporaneous outbreak therefore supported a clinical diagnosis of iatrogenic botulism. Botulism is principally a clinical diagnosis, and treatment should not be delayed while awaiting laboratory confirmation when clinical suspicion is sufficiently high [1,2].

Respiratory involvement represents the principal life-threatening complication of botulism. Descending paralysis may progress to involve the respiratory musculature, with severe cases requiring intubation and mechanical ventilation [1,2]. Normal oxygen saturation alone does not exclude evolving neuromuscular respiratory weakness, making serial neurological and respiratory assessment important [2]. Early involvement of critical care and difficult airway teams in this case facilitated serial forced vital capacity monitoring and contingency planning for respiratory deterioration. Despite prominent bulbar manifestations, the patient maintained her airway and did not require ventilatory support.

Botulism antitoxin neutralises circulating toxin that has not yet bound to neuronal receptors and therefore aims to prevent further neurological progression rather than immediately reverse established paralysis [1,2]. Evidence from foodborne botulism supports early antitoxin administration, although this evidence should not be directly extrapolated to quantify benefit in cosmetic iatrogenic disease [6]. UKHSA recommends that treatment of suspected iatrogenic botulism should not be delayed while awaiting laboratory investigations [1]. Antitoxin was therefore obtained and administered in this patient following specialist advice.

Neurological improvement occurred following antitoxin administration and was relatively rapid. However, a causal relationship between antitoxin and the subsequent improvement cannot be established from the temporal association alone. UKHSA guidance notes that recovery following antitoxin in iatrogenic botulism mat occur over a period ranging from several hours to several weeks [1]. The improvement observed in this patient therefore falls within the recognised clinical course, but it is not possible to distinguish the contribution of antitoxin from the natural recovery of the illness.

This case highlights an important practical issue regarding diagnostic sampling. UKHSA recommends obtaining serum before antitoxin administration when investigating suspected iatrogenic botulism [1]. In the present case, serum was collected only after antitoxin had been administered, reducing the opportunity for optimal laboratory confirmation. Even appropriately timed negative testing does not necessarily exclude iatrogenic botulism because circulating toxin concentrations may be low [1]. During the 2025 North East England outbreak, serum samples from three clinically defined cases were negative for BoNT [4]. Diagnostic serum should therefore be collected promptly before antitoxin whenever practicable, while ensuring that sample collection does not delay clinically indicated treatment.

Pyridostigmine 30mg four times daily was initially administered for symptomatic management. It was subsequently discontinued following specialist UKHSA advice that it was not recommended in this case. The decision was therefore based on specialist management advice rather than clinical deterioration or an adverse drug effect. The patient remained under observation following withdrawal and experienced no respiratory deterioration. Evidence regarding additional pharmacological treatments for botulism is limited, with current UKHSA guidance emphasising antitoxin, supportive care and symptomatic management in iatrogenic botulism and does not include pyridostigmine as standard therapy [7]. This aspect of the case highlights the value of specialist toxicology and public health involvement, particularly during an emerging outbreak.

The epidemiological context is particularly relevant. A detailed investigation of the 2025 North East England outbreak identified 25 cases following cosmetic BoNT-containing injections [4]. The investigation found an association between cases, particular practitioners, and an unlicensed BoNT-containing product. Laboratory analysis of seized product demonstrated a potency of 370 units per vial despite labelling indicating 200 units per vial [4]. National surveillance subsequently documented cases across five English regions [3]. The preparation, manufacturer, and dose administered to the patient in the present case were unknown; therefore, her illness cannot be attributed to a particular product or directly linked to products identified in the published outbreak investigation.

A recent scoping review of 113 reported cases of iatrogenic botulism found dysphagia in 82.3%, ptosis in 78.8%, generalised weakness in 65.5%, and respiratory failure in 12.4% [8]. Most patients ultimately recovered, although the clinical course varied [8]. Similarly, the major neurological abnormalities in the present patient had resolved by subsequent review, although residual nausea and reduced oral intake persisted. Improvement in the major neurological manifestations therefore did not immediately correspond to complete symptomatic recovery.

The principal limitations of this case are the absence of optimal laboratory confirmation, the lack of neurophysiological testing and the lack of information regarding the BoNT preparation, manufacturer, and administered dose. A causal association with a specific product implicated in the 2025 outbreak cannot therefore be established. Nevertheless, the temporal association with BoNT exposure, evolution of characteristic bilateral ocular and bulbar manifestations, absence of an identified alternative diagnosis, and contemporaneous epidemiological context supported the clinical diagnosis of iatrogenic botulism.

## Conclusions

Iatrogenic botulism should be considered in patients who develop progressive ocular, bulbar, or generalised neuromuscular symptoms following cosmetic BoNT administration, even when the initial clinical assessment is reassuring. In this case, the temporal relationship to BoNT exposure and subsequent evolution of characteristic ocular and bulbar manifestations supported a clinical diagnosis of suspected iatrogenic botulism. However, laboratory confirmation was unavailable, neurophysiological investigations were not performed, and alternative neurological diagnoses were not definitively excluded.

Suspected cases require close respiratory surveillance and early specialist toxicology and public health involvement. Serum for botulinum toxin testing should ideally be obtained before antitoxin administration, but diagnostic sampling should not delay treatment when clinical suspicion is high. This case highlights the importance of recognising an evolving neurological syndrome following cosmetic BoNT exposure and anticipating respiratory deterioration before it becomes clinically apparent.
